# Variational Characteristics of Vegetation Recovery Period Under Extreme Drought Across Various Land Cover Types in Guizhou Province, China

**DOI:** 10.1002/ece3.72869

**Published:** 2026-01-12

**Authors:** Yingqing Cen, Xu Xue, Jiamin Yang, Chuncan Meng

**Affiliations:** ^1^ Department of Ecology, College of Life Science Guizhou University Guiyang Guizhou Province China; ^2^ Key Laboratory of Plant Resource Conservation and Germplasm Innovation in Mountainous Region (Ministry of Education), Collaborative Innovation Center for Mountain Ecology & Agro‐Bioengineering (CICMEAB), College of Life Science/Institute of Agro‐Bioengineering Guizhou University Guiyang Guizhou Province China

**Keywords:** drought events, drought recovery time, Guizhou Province, local spatial cross‐correlation, MODIS

## Abstract

Drought, a recurring climatic challenge characterized by water scarcity, significantly affects the growth and functional stability of terrestrial vegetation. This study closely examines how extreme droughts vary and how the vegetation recovery period changes on the basis of multi‐source meteorological and remote satellite data. It also assesses the impacts of specific drought characteristics on vegetation recovery time in Guizhou Province, China. The results indicate that drought events are predominantly observed in the summer and autumn seasons across most of Guizhou Province, with a notable cessation of these dry spells often occurring in September and October. The northern and southwestern regions of Guizhou Province tend to experience the onset of drought conditions at an earlier date than other regions, with a concomitant trend of earlier termination of drought events in the northern locales. Droughts typically last 3–5 months, with mild and moderate droughts being the most common. The spatial distribution of vegetation recovery period from the droughts follows a discernible pattern, with most areas recovering within 2 months after drought ceases, whereas the Guiyang, Zunyi, Qiannan, and Qiandongnan regions need 4 to 6 months to recover in some years. Furthermore, substantial variations in vegetation recovery patterns have been observed among diverse land cover types. The average recovery period of forests ranged from 1.60 to 2.98 months, that of shrublands from 1.76 to 3.21 months, whereas the vegetation in croplands showed a relatively shorter recovery period, with most areas returning to normal levels within approximately 1 month. The recovery characteristics of vegetation were jointly influenced by the features of drought. Drought duration and severity often prolonged the length of the recovery period. This study provides a scientific basis for the formulation of regional drought management and ecological conservation strategies.

## Graphical abstract

Assessing characteristics of drought over the last 20 years in Guizhou Province, China. Unraveling the dynamics of vegetation recovery period across various land cover types. Drought duration and severity determine vegetation recovery period.

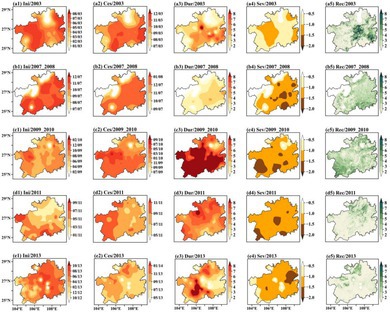

## 1 Introduction

Drought is a widespread natural disaster that exerts profound influences on both ecological and socio‐economic systems. It is primarily characterized by prolonged precipitation deficits and concurrent elevated temperature, which disrupt the balance between water supply and demand (Raposo et al. 2023). This extreme climatic event poses significant challenges to agricultural productivity and water resource management, while also threatening the stability and functioning of ecosystems (Meza et al. 2020; Dobson et al. 2020; Müller and Bahn 2022). Against the backdrop of global warming, research indicates that the frequency, intensity, and duration of drought events are on the rise in numerous regions, particularly in areas predisposed to drought, where global warming is intensifying the magnitude of its effects (Zarch et al. 2015; Zhang et al. 2023; Kim et al. 2020). Vegetation, an integral component of terrestrial ecosystems, performs a pivotal function in the exchange of matter and energy between the land and atmosphere through its growth dynamics and physiological activities (Jiang, Liu, Liu, et al. 2023; Jiang, Liu, and Xu 2023; Gong et al. 2022; Yang et al. 2022). Drought conditions are known to result in a depletion of soil moisture, a reduction in stomatal conductance, and a decline in the maximum photosynthetic capacity (Novick et al. 2016; Stocker et al. 2018; Luo and Keenan 2020). These alterations have the potential to impede transpiration and photosynthesis, which can result in a decline in plant biomass and a cessation in plant growth. Concurrently, these changes have the potential to diminish ecosystem productivity and exert a considerable influence on carbon cycling and climate regulation functions (Sippel et al. 2018).

Gross Primary Productivity (GPP) is a pivotal metric evaluating vegetation photosynthesis levels and ecosystem carbon sequestration capacity. It is frequently employed as an indicator in studies that examine the dynamic impacts of drought events on plant communities. For instance, research has demonstrated that grassland and agricultural regions typically undergo a decline in GPP during heatwaves and drought episodes (Zhu et al. 2021; Wu and Chen 2013). However, on a global scale, forests tend to demonstrate a comparatively diminished sensitivity to such extreme events (Flach et al. 2021). The aforementioned observations underscore the considerable diversity in the way ecosystems react to drought conditions.

In recent years, there has been an increase in scientific research focused on the characteristics of vegetation responses to drought stress. The responses exhibited by vegetation to periods of drought are influenced by a combination of factors, including the intensity and duration of the drought, the properties of soil moisture, and the specific climate conditions present in a given region (Bai et al. 2023; Torkaman Pary et al. 2024; Jiang, Liu, Liu, et al. 2023; Jiang, Liu, and Xu 2023; Yao et al. 2023; Zhan et al. 2022; Wang et al. 2024, 2025; Xue et al. 2024; Xue and Chen 2025). Research indicates that in semi‐arid ecosystems, grasslands manifest more prompt and pronounced responses to drought compared to croplands and deserts, whereas forest ecosystems exhibit comparatively muted reactions (Xu et al. 2021). Furthermore, within the same category of vegetation, forests and grasslands in tropical regions exhibit heightened sensitivity to anomalies in soil moisture in comparison to those in temperate and boreal regions, which can be attributed to the heightened vulnerability of tropical vegetation to drought conditions (Li, Piao, et al. 2023; Li, Zhang, et al. 2023).

Despite the abundance of research on vegetation responses to drought, studies focusing on vegetation recovery after drought stress cessation are limited. The capacity of vegetation to recover from an extreme drought is a fundamental metric for evaluating ecosystem resilience and adaptability (Smith et al. 2022; Oliver et al. 2015; Zhang et al. 2021). Preliminary research has indicated a potential positive correlation between the intensity and duration of drought events and the recovery periods of vegetation (Fathi‐Taperasht et al. 2022; Jiao et al. 2021). However, it is imperative to acknowledge that the specific length and patterns of recovery are also influenced by multiple factors, including ecosystem types and regional climatic conditions. For instance, vegetation recovery may require a longer time in areas with poor soil quality or insufficient water recharge capacity (Smith and Boers 2023). The duration of the recovery period is contingent on the growth stage and phenological phase of vegetation during the drought (Li, Piao, et al. 2023; Li, Zhang, et al. 2023), as the onset and cessation of drought events are closely related to vegetation phenology and growth cycles. However, extant research has focused primarily on the overall spatiotemporal characteristics of drought events, with insufficient detailed studies on the initiation and cessation of drought at the regional scale. Consequently, the refinement of regional analyses of drought initiation and cessation is imperative for enhancing our comprehension of vegetation response characteristics and recovery patterns under drought conditions.

Guizhou Province, located in southwestern China, is a typical ecologically fragile region characterized by karst landforms and complex terrain. The province's land cover is dominated by forests, shrublands, and croplands, which play a critical role in conserving water and soil as well as protecting biodiversity. However, the influence of the monsoon climate means that precipitation in this region is highly seasonal, mainly occurring in summer in the form of short periods of heavy rainfall (Zhang et al. 2010). Under karst geomorphological conditions, such precipitation rapidly infiltrates and is transferred laterally through subsurface conduits, making stable and usable water resources difficult to form (Le Mesnil et al. 2021). Furthermore, the risk of drought is not solely related to the total amount of precipitation; even in seasons with above‐normal rainfall, approximately 30%–50% of drought events can still be attributed to the heterogeneity of precipitation distribution (Wang et al. 2021). Long‐term monitoring of drought conditions in Guizhou indicates that summer droughts are common (Dai et al. 2023). Statistical analysis also reveals a high frequency and susceptibility of drought disasters in the province. Moderate droughts manifest approximately once every 3 years, severe droughts occur roughly once every 5 years, and extreme droughts, though less frequent, can still occur approximately once every decade (He et al. 2022; Cheng et al. 2018). Chronic stress reduces water availability and exerts sustained pressure on forests, shrublands, and croplands. This depresses their growth and compromises the regulatory and recovery capacities of ecosystems.

Previous studies have shown that analyzing extreme drought events within specific geographic regions is an effective way to understand their ecological impact. For example, research on the drought in 2009–2010 in Southwest China examined how vegetation responded and how the terrestrial carbon balance changed (Li et al. 2019), whereas studies on the 2022 drought in Guilin's karst region quantified ecosystem stress and vegetation degradation (Gao et al. 2025). Against this background, Guizhou Province was selected as a case study, and five extreme drought events that had notable impacts on GPP were identified from 2000 to 2021. This study aims to investigate how extreme droughts vary, how the recovery period of different land cover types (such as forests, shrublands, and croplands) changes, and how specific drought attributes influence these variations under extreme drought conditions in Guizhou Province, China. These analyses contribute to a more profound understanding of the dynamic changes in vegetation productivity in Guizhou Province and provide scientific evidence for the management and conservation of ecosystems in karst regions. Furthermore, the findings can serve as a point of reference for the development of drought adaptation strategies in other ecologically fragile regions.

## 2 Materials and Methods

### 2.1 Study Area

**FIGURE 1:**
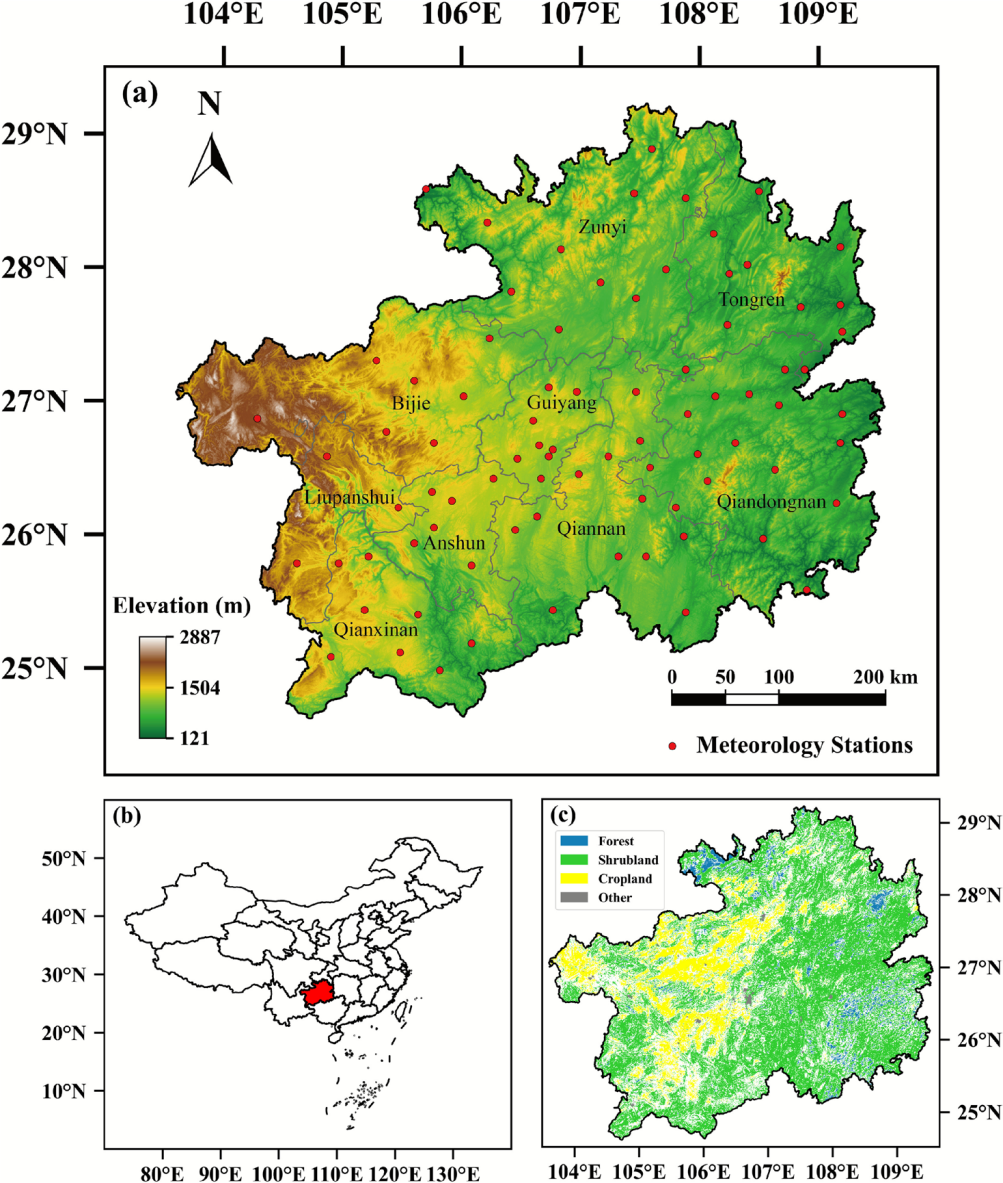
(a) Elevation (colors, unit: m) and locations (circles) of 83 national benchmark/basic meteorological stations in Guizhou Province. (b) Geographical location of Guizhou Province in China. (c) Spatial distribution of unchanged land cover type from 2001 to 2021. Forests encompass evergreen needleleaf forests, evergreen broadleaf forests, deciduous needleleaf forests, deciduous broadleaf forests, and mixed broadleaf and needleleaf forests. Shrublands include closed shrublands, open shrublands, woody savannas, and tropical savannas. Croplands encompass both croplands and cropland‐natural vegetation mosaics. Other types include permanent wetlands, urban and built‐up areas, ice and snow, barren lands, and water bodies.

Guizhou Province is located in southwestern China, with geographic coordinates ranging from 103°36′ E to 109°35′ E and 24°37′ N to 29°13′ N (see Figure 1b). The topography of Guizhou is characterized by a west‐high, east‐low gradient, dominated by mountains and hills, with an average elevation of approximately 1100 m (see Figure 1a). The western region is marked by the high terrain of the Wumeng Mountains, whereas the eastern region gradually transitions into the low hills of the Hunan and Hubei regions. The province is extensively characterized by the presence of karst landforms, which are indicative of a high degree of dissolution and erosional processes (Dai et al. 2023). These landforms profoundly influence the storage and flow of water resources. The subtropical monsoon climate of the province is marked by an average annual precipitation ranging from 1100 to 1400 mm, with significant variations across seasons. Rainfall is concentrated during the summer months, whereas the winter and spring seasons are relatively dry, resulting in an uneven distribution of water resources across different seasons (Chen et al. 2024). The province's land cover type is predominantly shrublands, with croplands primarily situated in Bijie and Anshun, and forests, which are comparatively sparse, more concentrated in the Zunyi, Tongren, and Qiandongnan areas (Figure 1c).

### 2.2 Datasets and Processing

The vegetation data used the MOD17A2H and MCD12Q1 products, which were provided by the Moderate Resolution Imaging Spectroradiometer (MODIS) satellite. The MODIS satellite, which was launched by the National Aeronautics and Space Administration (NASA), is designed for Earth observation and provides high‐frequency, high‐accuracy surface observation data. The MODIS satellite's applications encompass global change monitoring, vegetation growth, and climate change research. The MOD17A2H dataset provides GPP data for terrestrial ecosystems with a temporal resolution of 8 days and a spatial resolution of 500 m (https://appeears.earthdatacloud.nasa.gov/). To aggregate these data into a monthly‐scale dataset, a cumulative composite method was employed, thereby facilitating a more accurate representation of long‐term vegetation productivity changes. To minimize the impact of seasonal fluctuations and long‐term trends, the GPP time series was preprocessed in this study (Figure S1). Deseasonalization was achieved by subtracting the multi‐year mean value for each month, thereby weakening the seasonal effects caused by phenological patterns and climatic cycles. A linear detrending process was then performed on the deseasonalized series to remove long‐term trends (Schwalm et al. 2017). The resulting dataset encompasses a continuous 21‐year period, from 2000 to 2021. The MCD12Q1 dataset offers global land cover information for the period from 2001 to 2021, with a spatial resolution of 500 m and a temporal resolution of 1 year (https://appeears.earthdatacloud.nasa.gov/). In generating a map reflecting stable vegetation types, pixels with unchanged land cover types during this period were selected. This methodological decision was taken to eliminate the potential for bias resulting from alterations in land cover, thereby facilitating a more precise analysis of the impact of environmental factors, such as drought, on vegetation. Subsequently, the 17 land cover types present in the MCD12Q1 dataset were reclassified into four main categories: forests, shrublands, croplands, and other land types. These accounted for 4.94%, 77.52%, 16.76%, and 0.78% of the unchanged areas, respectively. This study focuses primarily on the analysis of the first three major land cover types.

The Standardized Precipitation Evapotranspiration Index (SPEI) was employed in the identification and analysis of the characteristics of drought events. SPEI is a multi‐scale drought index that combines precipitation and temperature data, effectively reflecting variations in hydrological and climatic droughts. Its extensive application in drought monitoring and assessment is well‐documented (Vicente‐Serrano et al. 2010; Dhangar et al. 2019; Rahman et al. 2021; Hua et al. 2017). Specifically, we utilized monthly precipitation and temperature data from 83 national benchmark/basic meteorological stations in Guizhou Province, which were made available by the National Meteorological Information Center of the China Meteorological Administration (http://cdc.cma.gov.cn/). These data span the period from 2000 to 2021, providing a reliable foundation for analyzing the temporal and spatial distribution characteristics of drought events in Guizhou Province. The R programming language and the Thornthwaite method were employed to calculate the SPEI index at a 3‐month time scale for each meteorological station (Thornthwaite 1948). The 3‐month time scale effectively captures the dynamic characteristics of drought events and is particularly suitable for analyzing short‐ to medium‐term drought dynamics (Zhou et al. 2019). Subsequently, on the basis of Python programming, we employed ordinary kriging interpolation to spatially interpolate the data on drought intensity, initiation and cessation, and duration to a resolution of 500 m × 500 m, aligning it with the resolution of the vegetation data (Goovaerts 1997; Isaaks and Srivastava 1989).

### 2.3 Methods

#### 2.3.1 Detection of Drought Events

**FIGURE 2:**
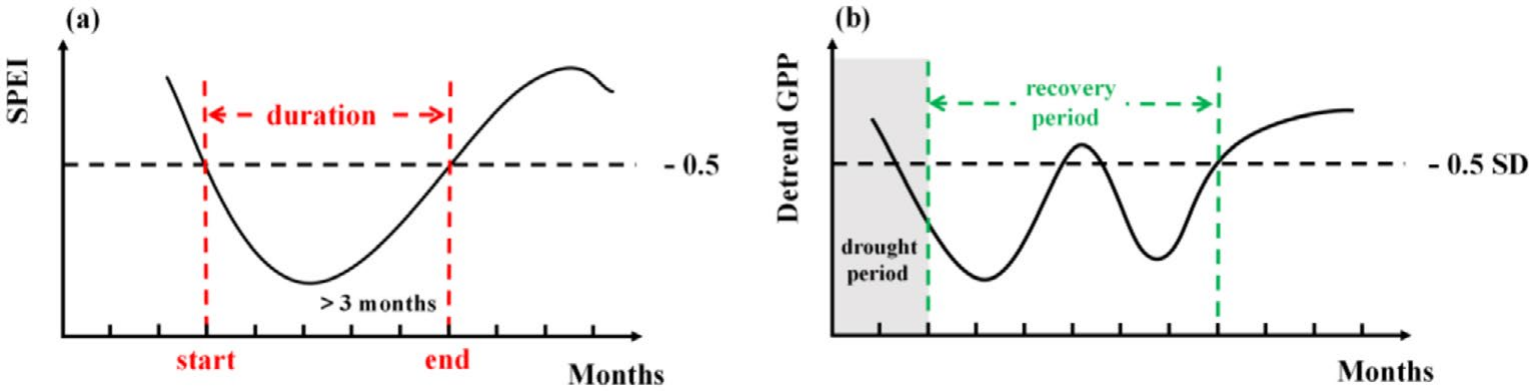
Definitions of (a) initiation, cessation, and duration of a drought event and (b) recovery period from drought.

The identification of extreme drought events in Guizhou Province was conducted through the implementation of the SPEI criteria, which stipulates that a regional mean SPEI value below −0.5 must persist for a minimum duration of 3 months. To assess the impact of these drought events on vegetation productivity, a selection of drought events with significant effects on GPP was further analyzed. This selection was based on integrating vegetation indices, which served as the foundation for the study. The classification of drought intensity followed the National Meteorological Drought Classification Standard (GB/T 20481‐2017), which divides drought intensity into four levels: mild drought (−1.0 < SPEI ≤ −0.5), moderate drought (−1.5 < SPEI ≤ −1.0), severe drought (−2.0 < SPEI ≤ −1.5), and extreme drought (SPEI < −2.0). The classification system is predicated on the deviation of SPEI values from normal climatic conditions, thereby representing the varying impacts of water deficits on ecosystems. The initiation of drought is defined as the first occurrence of an SPEI value below a threshold of −0.5, whereas the cessation of drought is marked by the recovery of SPEI above −0.5. The interval between these two points is indicative of the drought duration (Figure 2a). Utilizing these definitions, we proceeded to identify the spatiotemporal variations in drought intensity, initiation and cessation, and duration for extreme drought events in Guizhou Province. Furthermore, we integrated the province's three major land cover types—forests, shrublands, and croplands—to investigate the differential responses of each land cover type to extreme drought events. This approach was undertaken to elucidate the growth inhibition and adaptive characteristics of diverse land cover types under drought conditions.

#### 2.3.2 Definition of the Vegetation Recovery Period

Identifying the vegetation recovery period is a critical step in assessing the ecosystem resilience in the aftermath of extreme drought events. According to the framework proposed by Ingrisch and Bahn (2018), resilience is understood as a dynamic process that describes changes in ecosystem function following a disturbance. This process encompasses two core dimensions: resistance and recovery. Recovery refers to the process by which a system gradually returns to its pre‐disturbance baseline after being displaced from equilibrium. On the basis of this theoretical foundation, this study defines the recovery period as beginning at the conclusion of drought and ending when the deseasonalized and detrended GPP surpasses a threshold of −0.5 standard deviations (SD). Ensuring the robustness of the recovery period necessitates the establishment of a criterion, which stipulates that the deseasonalized and detrended GPP must maintain levels above −0.5 SD for a minimum duration of two consecutive months. The month marking the cessation of this period is designated as the recovery terminative point (refer to Figure 2b for illustration). Subsequent to the identification of the recovery period, a detailed analysis is conducted to examine the characteristics and patterns of recovery period variations among various land cover types within Guizhou Province under the stress of extreme drought conditions. To examine whether there were significant differences in recovery periods between different land cover types within the same drought event and between different drought events for each land cover type, the Kruskal–Wallis test was first employed to assess the overall significance of group differences. When the result of the overall test was significant (*p* < 0.05), Dunn's post hoc test was subsequently applied for pairwise comparisons, and the Bonferroni correction was used to control for multiple comparison errors.

#### 2.3.3 Local Spatial Cross‐Correlation

This study employed the Random Forest method and the Local Spatial Cross‐Correlation Index (LSCI) statistical approach to investigate the impact of drought characteristics on the recovery period following drought, considering different land cover types. Random Forest is an ensemble learning method that improves prediction accuracy by integrating the results of multiple decision trees, making it effective in handling non‐linear relationships (Bertrand et al. 2011; Gill et al. 2017). In this study, the number of trees was set to 300 to ensure model stability. The LSCI is a methodological framework that enables the analysis of local correlations among two or more variables within spatial datasets (Chen 2015). It possesses the capacity to reveal spatial variability and localized cross‐influences at different geographic locations, thus overcoming the limitations of conventional global correlation analysis. The relationship between drought variability characteristics and vegetation recovery period is subject to variation over time and may also be influenced by geographic location. Given that the impacts of drought conditions on vegetation recovery can differ across regions, traditional global correlation analysis methods may fail to capture these localized differences accurately. The LSCI method facilitates the execution of pixel‐by‐pixel correlation calculations, thereby unveiling the distinct relationships that exist between disparate locations within a region.

The following steps are imperative for the execution of this method: Initially, the raster data of the two variables is standardized to eliminate dimensional differences and ensure data comparability. Subsequently, employing the geographic coordinates of each pixel, the coordinates of its nearest neighbors are determined through the k‐nearest neighbor algorithm. Thereafter, a spatial weight matrix is constructed on the basis of the distances between neighboring pixels. The weight values for each row are then normalized to ensure that the sum of neighborhood weights for each pixel equals one. Subsequently, the correlation between each pixel and its neighbors is calculated by combining the spatial weight matrix, resulting in local cross‐correlation values that reveal the spatial correlation distribution between the two variables. Finally, by analyzing these correlation values, the differences in the relationships of vegetation recovery period with intensity and duration of drought across different regions are identified. This study ultimately selected *k* = 100 as the analysis parameter on the basis of a comparative analysis of different neighborhood scales and the results from random permutation tests repeated 999 times, followed by the False Discovery Rate (FDR) correction using the Benjamini–Hochberg method (Benjamini and Hochberg 1995).

## 3 Results

### 3.1 Identification of Drought Events

**FIGURE 3:**
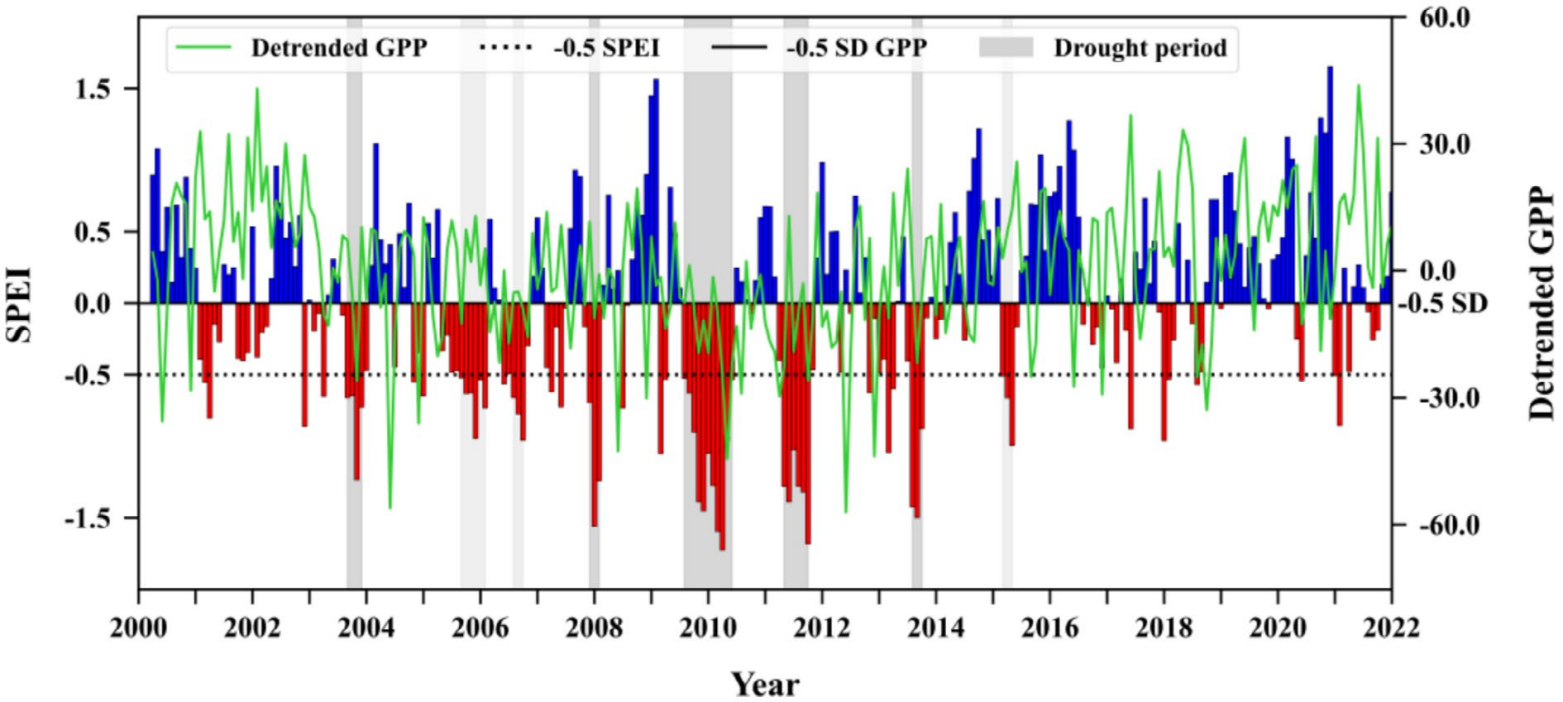
Regionally averaged SPEI‐3 time series for Guizhou Province from 2000 to 2021. The green line indicates the deseasonalized and detrended GPP values, whereas the gray shaded areas denote drought periods. The dark gray regions highlight the selected drought events.

According to the established criteria, eight drought events were identified in Guizhou Province from 2000 to 2021. Among these, five drought events that had a notable negative impact on GPP were selected as the focus of the present study. These drought events were demarcated by dark gray shading in Figure 3. Of these, the droughts from July 2009 to May 2010 and from April to September 2011 were the most severe, characterized by prolonged durations and very low SPEI values, indicative of extreme drought. Despite their comparatively brief duration, the drought events spanning from August to November 2003, November 2007 to January 2008, and July to September 2013 also manifested as periods of water scarcity, as suggested by their persistently low SPEI values. The GPP during these periods exhibited substantial declines, underscoring the assertion that even shorter‐lived drought events, if sufficiently intense, can exert pronounced detrimental effects on local ecosystems.

**FIGURE 4:**
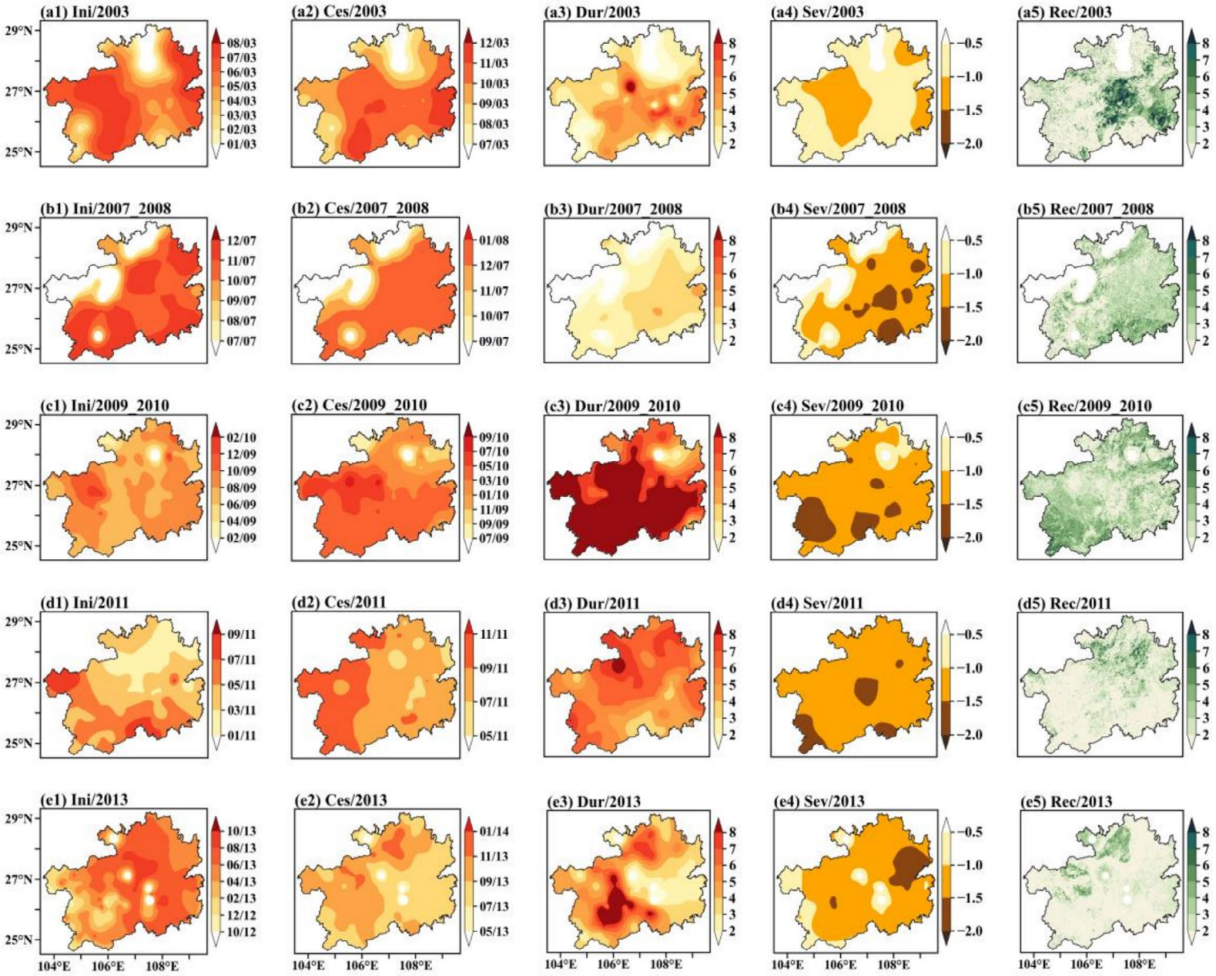
Spatial characteristics of the five drought events, including (a1, b1, c1, d1, e1) initiation and (a2, b2, c2, d2, e2) cessation timing, (a3, b3, c3, d3, e3) duration, (a4, b4, c4, d4, e4) severity, and (a5, b5, c5, d5, e5) vegetation recovery period for the drought of (a1–a5) 2003, (b1–b5) 2007–2008, (c1–c5) 2009–2010, (d1–d5) 2011, and (e1–e5) 2013, respectively. Both duration and recovery time are expressed in months.

### 3.2 Spatial Distributions of Drought Events and Vegetation Recovery Period

Guizhou Province experienced five distinct drought events (e.g., the year of 2003, 2007, 2009, 2011, and 2013) between 2000 and 2021, each exhibiting significant differences in initiation and cessation times, affected areas, and intensity.

The analysis reveals that the initiation and cessation times of droughts exhibit significant seasonal patterns and spatial variation. During the 2003, 2009, and 2013 droughts, most droughts began in summer; however, during the 2007 drought, most began in the autumn, and during the 2011 drought, most began in spring (see Table 1). Droughts also generally end in autumn in most regions, although the 2009 drought was more persistent, lasting until spring the following year. Additionally, two special types of spatiotemporal patterns in the initiation and cessation of droughts were identified. One type is synchronous across the entire region, as seen in the 2007 event, where droughts occurred and ended almost simultaneously across the province (Figure 4b1,b2). The other type is progressive, as seen in the 2011 drought, which gradually advanced from north to south. Its cessation also exhibited east‐to‐west variation, ending earlier in the eastern regions and later in the western ones (Figure 4d1,d2).

**TABLE 1:** Dominant seasons of drought initiation and cessation with their corresponding area proportions, along with the average drought duration, severity, and recovery period. Both duration and recovery time are expressed in months.

Events	Dominant season of initiation	Dominant season of cessation	Duration	Severity	Recovery
2003.08–2003.11	Summer (60.31%)	Autumn (88.00%)	3.75	−0.91	2.79
2007.11–2008.01	Autumn (91.64%)	Winter (90.30%)	2.70	−1.24	2.92
2009.07–2010.05	Summer (59.17%)	Spring (58.31%)	8.44	−1.32	3.12
2011.04–2011.09	Spring (62.53%)	Autumn (77.90%)	5.91	−1.41	1.97
2013.07–2013.09	Summer (51.29%)	Autumn (71.93%)	4.68	−1.23	1.74

**FIGURE 5:**
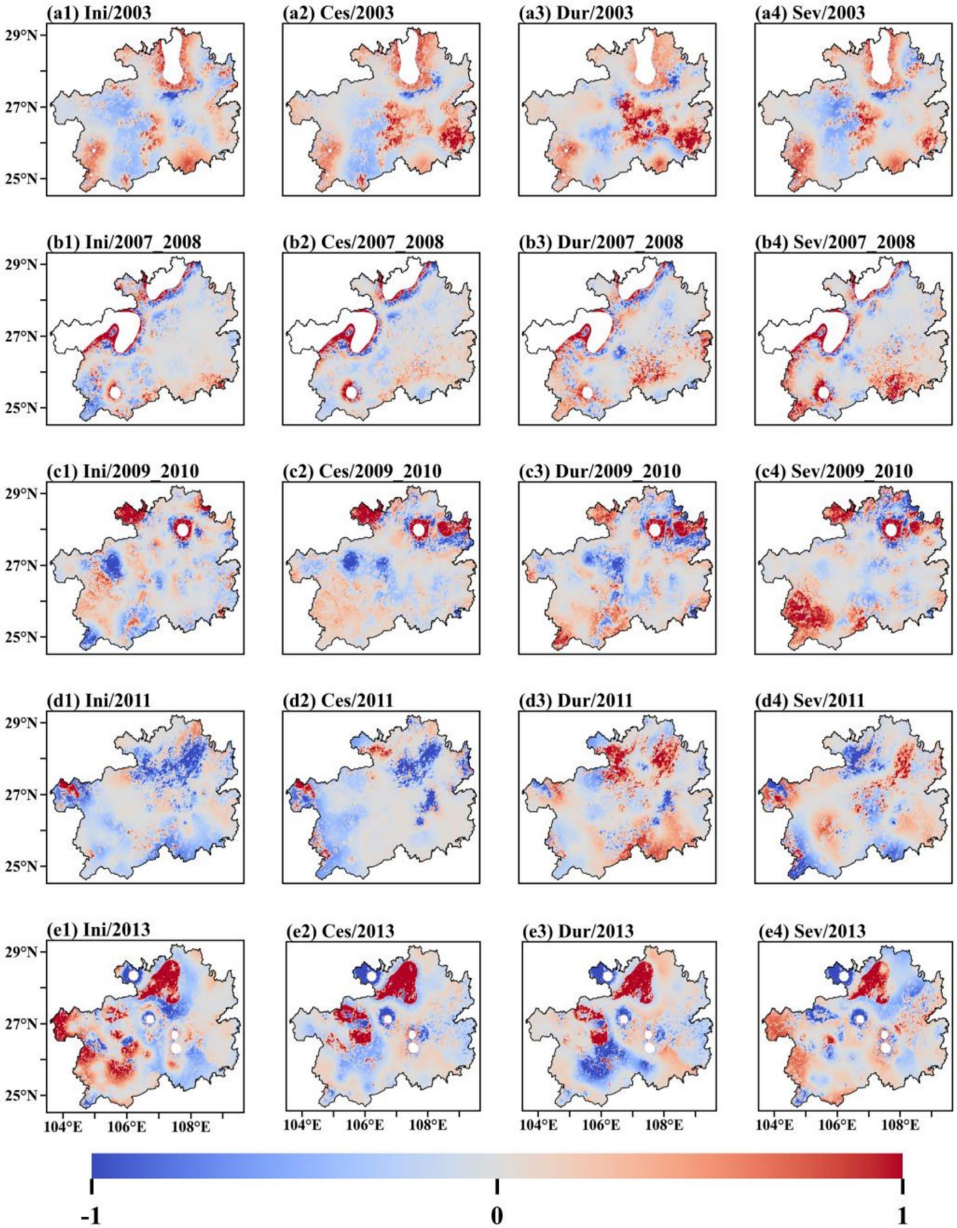
Local spatial correlations between drought characteristic indicators and the recovery period, including the correlations between the recovery period and the timing of initiation and cessation, the duration and severity of the drought events for (a1–a4) 2003, (b1–b4) 2007–2008, (c1–c4) 2009–2010, (d1–d4) 2011, and (e1–e4) 2013, respectively. Correlation values between 0 and 1 indicate positive correlations, whereas values between −1 and 0 indicate negative correlations.

In terms of duration and intensity, droughts in Guizhou Province typically lasted for 3–5 months. However, some regions experienced prolonged impacts, with areas such as Bijie, Anshun, Qianxinan, and Qiannan frequently experiencing extreme droughts lasting up to 8 months. These areas are therefore considered to be at high risk of prolonged drought (Figure 4d3,e3). Three categories of drought intensity were observed: mild, moderate, and severe. Moderate droughts were the most common and had the most extensive impact. Regions such as Anshun, Qianxinan, Qiannan, Qiandongnan, and Tongren were affected by severe droughts across multiple events (Figure 4b4,c4,d4,e4).

The average recovery time for the entire province after a drought was approximately 1–3 months (Table 1). During the 2011 and 2013 droughts, 78.02% and 83.27% of affected areas, respectively, returned to normal levels within 2 months (Figure 4d5,e5). However, during the 2003, 2007, and 2009 droughts, most regions required longer recovery times. Notably, areas such as Qiannan, Qiandongnan, and Tongren required recovery periods of 6 months or more in multiple events (see Figure 4a5,b5,c5).

A comprehensive analysis of the five drought events that have occurred in Guizhou Province over the past two decades disclosed distinct seasonal characteristics in terms of the initiation and cessation of droughts, with initiation typically occurring in summer and cessation primarily occurring in autumn. Recurrent hotspots of drought duration and intensity were identified in regions such as Anshun, Qianxinan, and Qiannan. In terms of recovery periods, most areas recovered within 3 months. However, certain regions, including Qiannan, Qiandongnan, and Tongren, exhibited delayed recovery and require special attention.

### 3.3 Relationship of Drought Feature With the Vegetation Recovery Period

Guizhou Province has experienced five distinct drought events between 2000 and 2021, and the spatial distribution of the vegetation recovery period has exhibited notable variations over time. During the 2003 drought event, the recovery period was most strongly influenced by the drought initiation time (Figure S3a). Spatially, the recovery period showed a positive correlation with the drought initiation time in Zunyi but a negative correlation in Bijie and Anshun. It was also positively correlated with the drought cessation time, duration, and severity in Guiyang, Qiannan, and Qiandongnan (Figure 5a2–a4). Conversely, a weak negative correlation was observed in Bijie and Anshun (Figure 5a1–a4). This suggested that under the 2003 drought conditions, the regions where drought started early and concluded late exhibited a prolonged duration and heightened severity of drought, resulting in an extended recovery period. Conversely, the region where drought conditions commenced and concluded late exhibited a shorter duration and milder severity, leading to reduced damage to vegetation and a subsequent accelerated recovery process.

For the 2007 drought, the recovery period was most strongly influenced by drought severity (Figure S3a). Spatially, the recovery period exhibited a strong positive correlation with the duration and severity of the drought in the Qianxinan and Qiannan regions, where the recovery period persisted generally over 4 months (Figure 5b3,b4). For the 2009 drought, the recovery period had a strong positive correlation with the duration and severity of the drought in the Qianxinan regions (Figure 5c3,c4). Prolonged duration and high severity of the 2007 and 2009 drought exacerbated vegetation damage and necessitated a more extended period for recovery. For the 2011 drought, the recovery period exhibited a strong positive correlation with the duration and severity, whereas a noteworthy negative correlation with the initiation and cessation timing of the drought in the Tongren regions (Figure 5d1–d4). This indicates prolonged duration and high severity of the drought extended the recovery period. For the 2013 drought, a prolonged recovery period in the Zunyi region was closely positively correlated with the initiation and cessation timing, the duration, and severity of the drought (Figure 5e1–e4).

The above results indicate that the recovery period across Guizhou Province was primarily influenced by drought severity (Figure S3b). Although drought duration and the recovery period exhibit a generally consistent spatial correlation, drought severity contributes synergistically to the extension of the recovery period, the interactions among drought characteristics exert a complex influence on recovery time.

### 3.4 Variational Characteristics of the Recovery Period Across Various Land Cover Types

**FIGURE 6:**
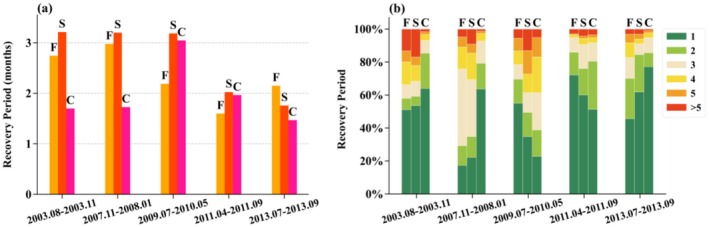
(a) Post‐drought recovery time and (b) recovery duration percentage required by different land cover types. Here, F represents forests, S represents shrublands, and C represents croplands.

The recovery period exhibited by various land cover types following exposure to drought conditions manifests specific patterns and variability. Significant differences in response to drought and recovery period are generally observed between land cover types across different drought events and within the same event (Figures S4–S6). Specifically, the overall trend indicates that the average recovery period for each land cover type is approximately 1–3 months, with shrublands generally requiring the longest recovery time and croplands exhibiting the shortest recovery period (Figure 6a, Figure S2). The average recovery period for forests ranged from 1.60 to 2.98 months. The longest recovery period was observed following the 2007 drought, which lasted 2.98 months, whereas the shortest recovery period required only 1.60 months under the 2011 drought (Figure 6a). Specifically, forests required a longer period with greater than 3 months accounting for 70.75% to recover from the 2007 drought (Figure 6b), and recovered more quickly under the 2003, 2009, 2011, and 2013 droughts, with a recovery time of less than 2 months accounting for 57.93%, 69.58%, 85.91%, and 70.03%, respectively (Figure 6b). The average recovery period for shrublands ranged from 1.76 to 3.21 months. The shrubland required a longer recovery period under the 2003, 2007, and 2009 droughts, with those greater than 3 months accounting for 40.81%, 65.23%, and 50.55%, respectively (Figure 6a,b), and recovered more quickly with less than 2 months accounting for 76.07% and 84.49% under the 2011 and 2013 droughts. For the cropland, vegetation required a longer average recovery period of 3.05 months, with those longer than 3 months accounting for 61.18% under the 2009 drought, and recovered more quickly with less than 2 months accounting for 85.38%, 79.24%, 80.43%, and 85.60% under the 2003, 2007, 2011, and 2013 droughts, respectively.

**FIGURE 7:**
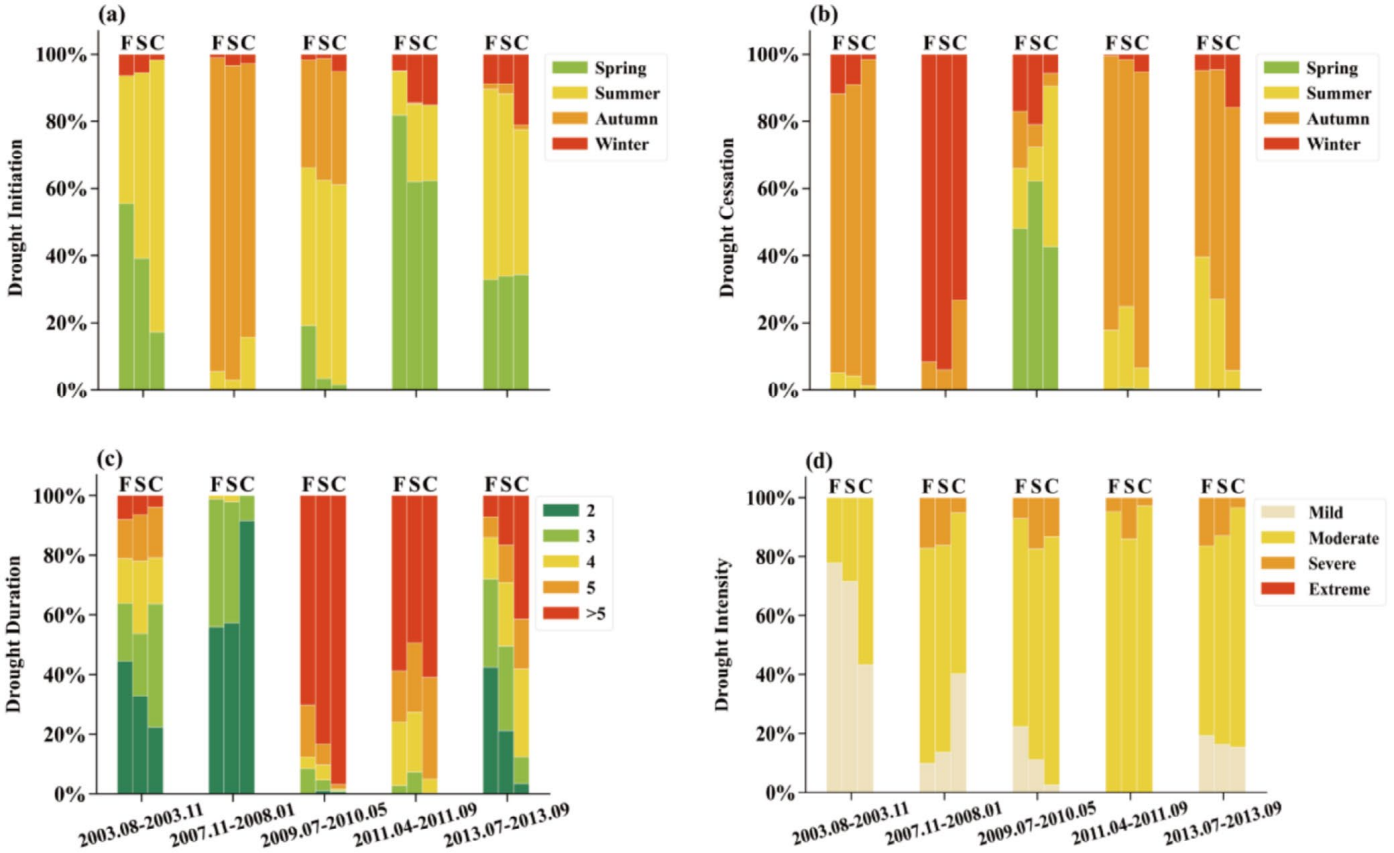
Drought conditions across different land cover types, including (a) the percentage of drought initiation, (b) the percentage of drought cessation, (c) the percentage of drought duration, and (d) the percentage of drought severity.

Furthermore, we also explored the factors influencing the differences in recovery periods among varied land cover types. The results indicate that, during the 2003 drought event, the recovery period was most strongly influenced by the initiation time of the drought, followed by its duration and cessation time (Figure S3a). In areas dominated by forests and shrubs, droughts typically began in the early growing season (spring) and ended in autumn, spanning almost the entire growing period (Figure 7a,b). Such prolonged stress during the critical stage of vegetation physiological activity exerts a stronger impact on the recovery process.

The 2007 drought event primarily occurred in autumn, with a relatively short duration, and mostly ended in winter (Figure 7a,b). In most regions, the drought lasted for 2–3 months, with overall severity dominated by moderate drought, whereas some areas reached the level of severe drought (Figure 7c,d). The impact of this drought on forestland and shrubland recovery was relatively unique, which may be related to the timing of the drought and the compounding effect of subsequent extreme climatic events. The drought occurred in late autumn and was immediately followed by a large‐scale snow disaster in early 2008. Low temperatures and snowfall may have caused damage to tree branches and leaves, further delaying vegetation recovery (Bragg et al. 2003; Wang et al. 2019). In contrast, the faster recovery of cropland may be attributed to the relatively mild drought experienced by cropland, where areas with a drought duration of 2 months accounted for as much as 91.59%, and the proportion of mildly drought‐affected areas was significantly higher than that of forests and shrublands, with a smaller extent of severely drought‐affected regions. In addition, agricultural management measures such as irrigation and tillage may have also accelerated the recovery process and further promoted crop growth.

The 2009 drought event was the longest‐lasting drought during the study period, with drought persisting for more than 5 months in most regions and exhibiting high severity (Figure 7c,d). This drought primarily occurred in summer and autumn and mostly ended in the spring of the following year, resulting in a prolonged water deficit and exerting a profound impact on vegetation (Figure 7a,b). The recovery periods of forests, shrublands, and croplands were all relatively long.

During the 2011 drought event, most regions experienced prolonged impacts of relatively high severity lasting over 4 months, yet the overall vegetation recovery period was relatively short. Notably, when this drought occurred, vegetation in some parts of Guizhou Province was still recovering from the 2010 drought and had not fully returned to normal levels (Figure 4c2,c5,d1). This suggests that under consecutive drought stress, the vegetation recovery process may exhibit more complex dynamics.

The 2013 drought event primarily occurred in spring and summer and ended in autumn (Figure 7a,b), with a duration mostly ranging from 2 to 5 months (Figure 7c). There were differences in drought severity experienced by different land cover types, among which the proportion of forests affected by severe drought was relatively high, reaching 16.41%, whereas the proportion of severely drought‐affected areas in cropland was relatively low, at only 3.54% (Figure 7d). The combination of drought duration and severity may have influenced the recovery rates of different land cover types to some extent. As a result, the recovery period of forests was relatively long, whereas the recovery of cropland was faster, with most areas able to achieve a rebound in productivity within 2 months.

Overall, there is a certain positive correlation between drought duration and severity and the vegetation recovery period, with longer and more severe droughts often accompanied by longer recovery periods. In addition, the seasonal characteristics of drought occurrence may also influence the recovery rate of vegetation to some extent. For example, drought events occurring in autumn and winter may delay vegetation recovery under conditions of low temperature and reduced precipitation. Differences also exist in the recovery performance among different land cover types. Forests and shrublands usually recover more slowly, especially when experiencing high‐severity drought. In contrast, the recovery of cropland is more easily regulated and influenced by human activities.

## 4 Discussion

The spatiotemporal distribution of drought events in Guizhou Province and their impacts on vegetation vary across different regions and land cover types, which is consistent with previous studies. This study found that drought events in Guizhou Province generally occur in summer and mostly end in autumn. In terms of duration, the southwestern region of Guizhou often experiences long‐term drought, whereas the Qiannan and Qiandongnan regions are more prone to high‐severity drought. These phenomena may be related to abnormal atmospheric circulation and the unique karst topography (Xiao et al. 2019; Feng et al. 2014; Liu et al. 2015; Cheng et al. 2018).

In terms of responses among different land cover types, we revealed the differences in drought duration, severity, and recovery period among forests, shrublands, and croplands. Forests generally experienced shorter drought durations and, compared to shrublands and croplands, had a higher proportion of mildly drought‐affected areas. This may be related to their strong water regulation capacity and well‐developed deep root systems, which can alleviate physiological stress caused by drought to some extent (Li et al. 2024; Jiao et al. 2021). Despite their strong drought resistance, forests do not always show an advantage in post‐drought recovery ability. Some studies have pointed out that forests may have relatively longer recovery periods. However, the results of this study show that the average recovery period of shrublands is longer, which differs from previous conclusions (Shao et al. 2024; Han et al. 2024; Zhang et al. 2021). This phenomenon may be due to the thin soil layers and limited water storage capacity in karst areas, which easily lead to the “dry‐wet alternation effect.” The drought resistance of shrublands is not as strong as that of forests, making them more susceptible to secondary stress due to insufficient moisture during the post‐drought recovery process (Tian et al. 2024). In addition, shrublands are widely distributed in Guizhou Province and are easily affected by various uneven environmental factors, which may further complicate and destabilize their recovery process. For example, as cropland is mainly distributed in the cities of Bijie, Anshun, Liupanshui, Qianxinan, Zunyi, and Guiyang (Figure 1c), we examined the response and recovery characteristics of cropland and shrubland in these six regions. The results showed that the overall patterns in these areas were generally consistent with the provincial‐level trends (see Figure 7, Figure S7a–d). However, focusing only on these six regions revealed that the recovery period of shrubland was shorter in 2003 and 2007 (Figure S7e,f). Notably, the results show that the average recovery period of shrublands gradually shortened across the province during the study period, decreasing from 3.21 months in 2003 to 1.76 months in 2013. Since the early 21st century, the frequency and intensity of drought events in Guizhou Province have increased (Cheng et al. 2018). The shortening of shrub recovery periods observed may reflect structural or physiological adjustments that reduce water loss and improve water use efficiency in the face of recurrent long‐term drought. This, in turn, alleviates physiological damage during drought episodes (Frank et al. 2015; Marchin et al. 2022; Ozturk et al. 2021; Flach et al. 2021; Tangjialeke et al. 2024). Additionally, Xue et al. (2023)'s research indicates that extreme temperature values in Guizhou Province increased significantly between 1960 and 2019, revealing an overall warming trend. Furthermore, warming can enhance shrub growth performance (Wu et al. 2025; Zamin et al. 2014). Conversely, one study suggests that vegetation change is primarily driven by human‐led ecological restoration projects, with climate factors contributing relatively less at around 24% (Wei et al. 2021). Therefore, the shortening of shrub recovery periods may result from a combination of prolonged drought stress, rising regional temperatures, changes in other climate variables, and increased investment in ecological management. In contrast, the recovery period of cropland is usually shorter, but it is significantly prolonged when experiencing long‐duration drought events. This may be related to soil structure degradation and soil moisture deficit. Prolonged drought may not only lead to soil compaction but also limit the availability of irrigation resources, thereby imposing certain constraints on agricultural management activities, which in turn affects the vegetation recovery process (Cruz et al. 2021; Bonanomi et al. 2020; Luo et al. 2019; Xu et al. 2020). In addition, the duration and severity of droughts show a positive correlation with the recovery period. However, if vegetation experiences a new drought before it has fully recovered from the previous one, the previous drought experience may have created a memory of stress within the vegetation. This enables it to activate defense mechanisms more rapidly under subsequent drought stress, thereby improving its response efficiency to environmental stress (Jacques et al. 2021). Therefore, although GPP still fluctuated during the 2011 drought, the overall decline was relatively small and remained at a relatively stable level (Figure 3). Moreover, the seasonal characteristics of drought also influence the speed of vegetation recovery to some extent. If a drought occurs during the early to middle stages of the growing season, when vegetation is in a phase of high water demand and active carbon absorption, drought stress can substantially impact ecosystem productivity. If the drought persists into autumn and winter, the carbon reserves accumulated earlier period will gradually be depleted. Meanwhile, reduced precipitation and insufficient soil moisture replenishment in Guizhou further impede the recovery of physiological functions, thereby prolonging the recovery period (Xiao et al. 2025; Lian et al. 2020).

Although this study revealed the spatiotemporal distribution characteristics of typical drought events in Guizhou Province over the past two decades and their impacts on the recovery periods of different land cover types, some aspects still require further investigation. First, the identification of the recovery period currently relies mainly on the changing trend of GPP. Although this effectively reflects the dynamic changes in vegetation productivity, it does not incorporate other indicators such as Leaf Area Index, Enhanced Vegetation Index, or the fraction of Absorbed Photosynthetically Active Radiation, which may, to some extent, limit the understanding of the multidimensional recovery process of vegetation status and function. In addition, our analysis of the factors influencing the recovery period primarily examined the correlation between drought characteristics and the recovery period. However, we occasionally observed a negative correlation between drought duration/intensity and the recovery period, suggesting that other factors may also influence recovery. We have not yet systematically accounted for potential drivers such as hydrothermal conditions, soil properties, or human interventions. A more in‐depth investigation into the roles of soil water storage and availability, and their relationship with atmospheric drought in vegetation recovery mechanisms, is also an important direction for future research (Vicca et al. 2012; Knapp et al. 2023).

Meanwhile, although the drought identification criterion adopted in this study is generally reasonable and effective, it may homogenize the impact of short‐duration, high‐intensity droughts. Pulse (flash) droughts are characterized by rapid intensification and often occur alongside elevated vapor pressure deficit and temperature, amplifying stress. Under these conditions, vegetation tends to exhibit lower resistance (Otkin et al. 2018; Zhang et al. 2025). Therefore, our results reflect the aggregate impacts of such events, but do not isolate their specific effects. Accordingly, future research could integrate multi‐source remote sensing and climatic data to differentiate between types of droughts and thereby improve our understanding of the mechanisms behind vegetation recovery.

## 5 Conclusion

This study, on the basis of the SPEI index and GPP index, analyzed the spatiotemporal characteristics of five typical drought events in Guizhou Province from 2000 to 2021 and examined the response and recovery characteristics of three major land cover types under different drought conditions. The results showed that droughts in Guizhou Province generally occurred in summer and mostly ended in autumn. The duration was typically between 3 and 5 months, with some areas experiencing droughts lasting over 8 months. The severity was mainly characterized by moderate drought, whereas the frequency of severe droughts was higher in the Qiannan region. Recovery period analysis indicated that most areas recovered within 2 months after the end of drought, whereas some regions in the Qiannan, Guiyang, and Zunyi regions showed delayed recovery, with certain years extending to 4–6 months. In addition, vegetation recovery characteristics after drought varied significantly among different land cover types: forests had an average recovery period between 1.60 and 2.98 months, shrublands between 1.76 and 3.21 months, and croplands showed relatively shorter recovery, with most areas recovering in about 1 month. Overall, among the various drought characteristics affecting the recovery period, the recovery period is most strongly influenced by drought severity, followed by drought initiation time, duration, and cessation time. Different land cover types exhibited different characteristics in this relationship: shrublands were more sensitive to changes in drought severity, showing longer recovery periods under relatively higher drought severity, whereas the recovery period of cropland was more affected by drought duration, with significantly slower recovery under long‐term drought conditions.

Our study contributes to revealing the spatiotemporal response differences and recovery characteristics of different land cover types under drought, providing a scientific basis for understanding vegetation resilience and for drought risk management.

## Author Contributions

**Yingqing Cen:** conceptualization (supporting), methodology (equal), visualization (lead), writing – original draft (lead), writing – review and editing (supporting). **Xu Xue:** conceptualization (lead), methodology (equal), supervision (lead), writing – review and editing (lead). **Jiamin Yang:** methodology (equal). **Chuncan Meng:** methodology (equal).

## Funding

This work was supported by Guizhou Provincial Basic Research Program (Natural Science) (Grant No. QianKeHeJiChu–ZK[2024]YiBan025) and National Natural Science Foundation of China (Grant No. 42465003).

## Conflicts of Interest

The authors declare no conflicts of interest.

## Supporting information


**Figure S1:** Monthly mean GPP and detrended GPP time series in Guizhou Province from 2000 to 2021.
**Figure S2:** Comprehensive distribution characteristics of recovery periods across different land cover types.
**Figure S3:** Importance of drought characteristics for the recovery period on the basis of the Random Forest (%IncMSE).
**Figure S4:** Significance results of recovery period differences among drought events for the same land cover type (Kruskal–Wallis and Dunn tests).
**Figure S5:** Significance results of recovery period differences among land cover types within the same drought event (Kruskal–Wallis and Dunn tests).
**Figure S6:** Significance results of differences in drought characteristics (initiation, cessation, duration, and severity) among land cover types within the same drought event (Kruskal–Wallis and Dunn tests).
**Figure S7:** Drought conditions across different land cover types, including (a) the percentage of drought initiation, (b) the percentage of drought cessation, (c) the percentage of drought duration, (d) the percentage of drought severity, (e) post‐drought recovery time, and (f) recovery duration percentage required by different land cover types.

## Data Availability

The data that support the findings of this study are openly available at https://doi.org/10.5061/dryad.3j9kd51wx and https://appeears.earthdatacloud.nasa.gov/.
