# Supplementary material for: Variational Characteristics of Vegetation Recovery Period Under Extreme Drought Across Various Land Cover Types in Guizhou Province, China
Source: Ecol Evol. 2026 Jan 12;16(1):e72869. doi: 10.1002/ece3.72869 (PMC12795616; doi:10.1002/ece3.72869)
Supplement: Supplementary file 1 — Figure S1: Monthly mean GPP and detrended GPP time series in Guizhou Province from 2000 to 2021. Figure S2: Comprehensive distribution characteristics of recovery periods across different land cover types. Figure S3: Importance of drought characteristics for the recovery period on the basis of the Random Forest (%IncMSE). Figure S4: Significance results of recovery period differences among drought events for the same land cover type (Kruskal–Wallis and Dunn tests). Figure S5: Significance results of recovery period differences among land cover types within the same drought event (Kruskal–Wallis and Dunn tests). Figure S6: Significance results of differences in drought characteristics (initiation, cessation, duration, and severity) among land cover types within the same drought event (Kruskal–Wallis and Dunn tests). Figure S7: Drought conditions across different land cover types, including (a) the percentage of drought initiation, (b) the percentage of drought cessation, (c) the percentage of drought duration, (d) the percentage of drought severity, (e) post‐drought recovery time, and (f) recovery duration percentage required by different land cover types. [file ECE3-16-e72869-s001.docx]

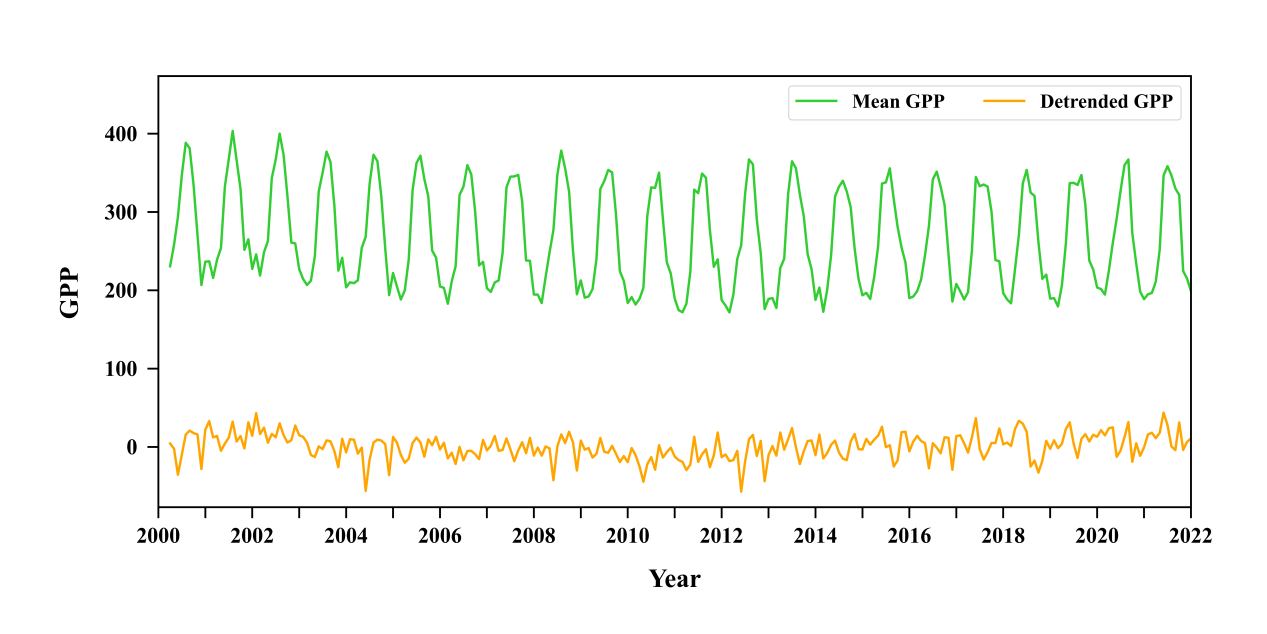


**Figure S1** Monthly mean GPP and detrended GPP time series in Guizhou Province from 2000 to 2021. The green line represents the monthly mean GPP, and the orange line represents the detrended and deseasonalized GPP values.


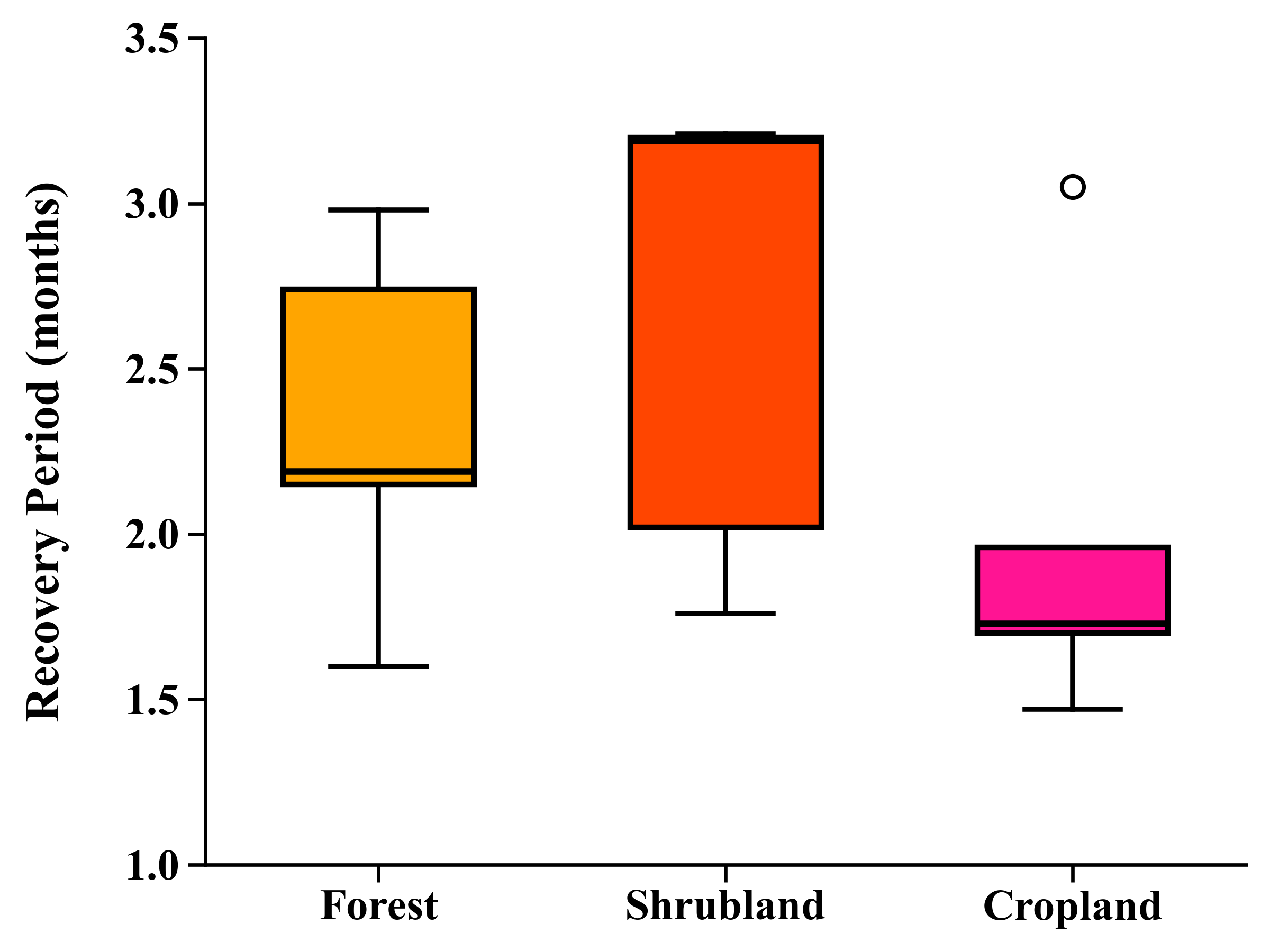


**Figure S2** Comprehensive distribution characteristics of recovery periods across different land cover types.


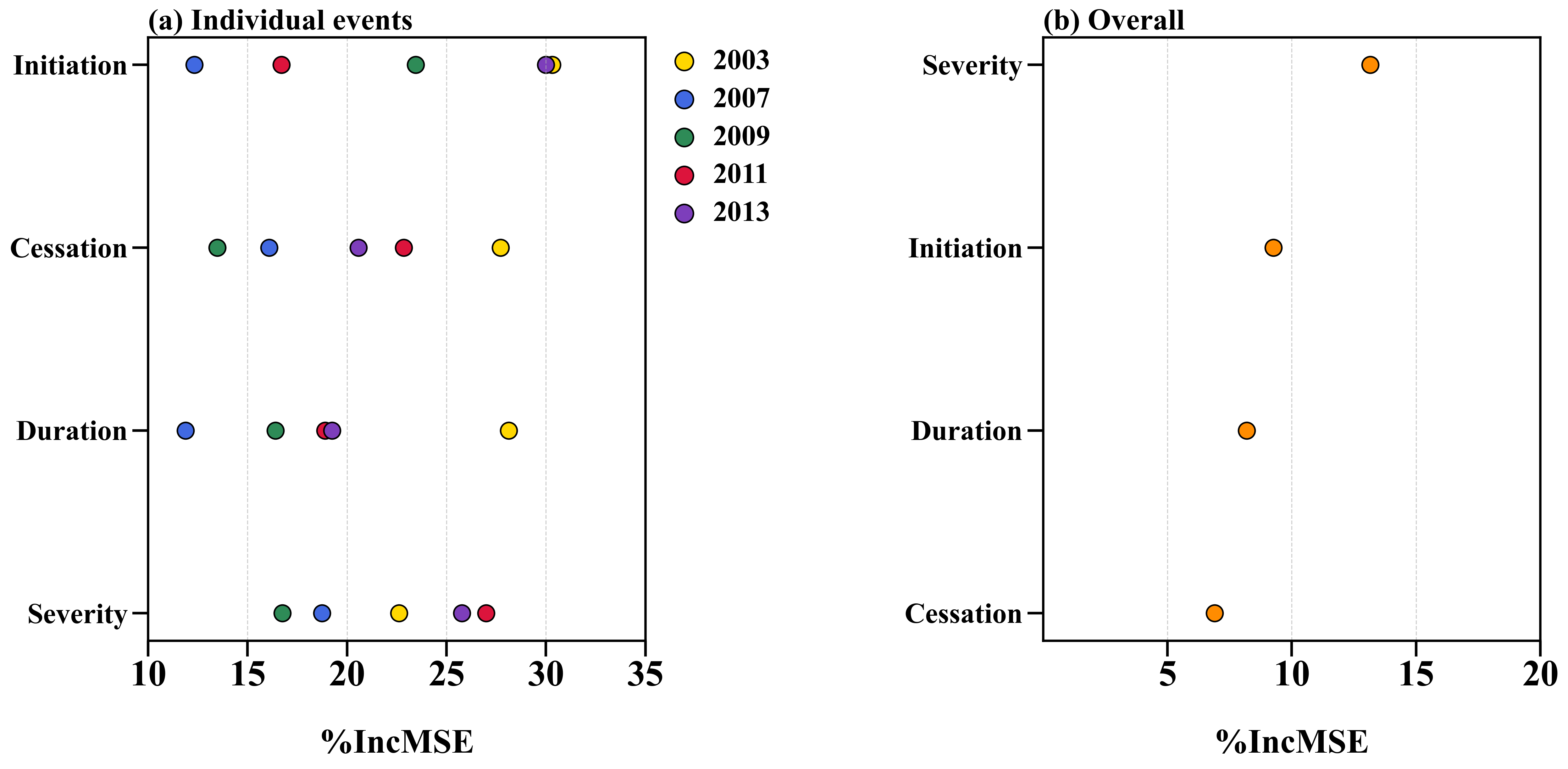


**Figure S3** Importance of drought characteristics for the recovery period based on the Random Forest (%IncMSE). (a) Importance results for individual events; (b) Overall importance results.


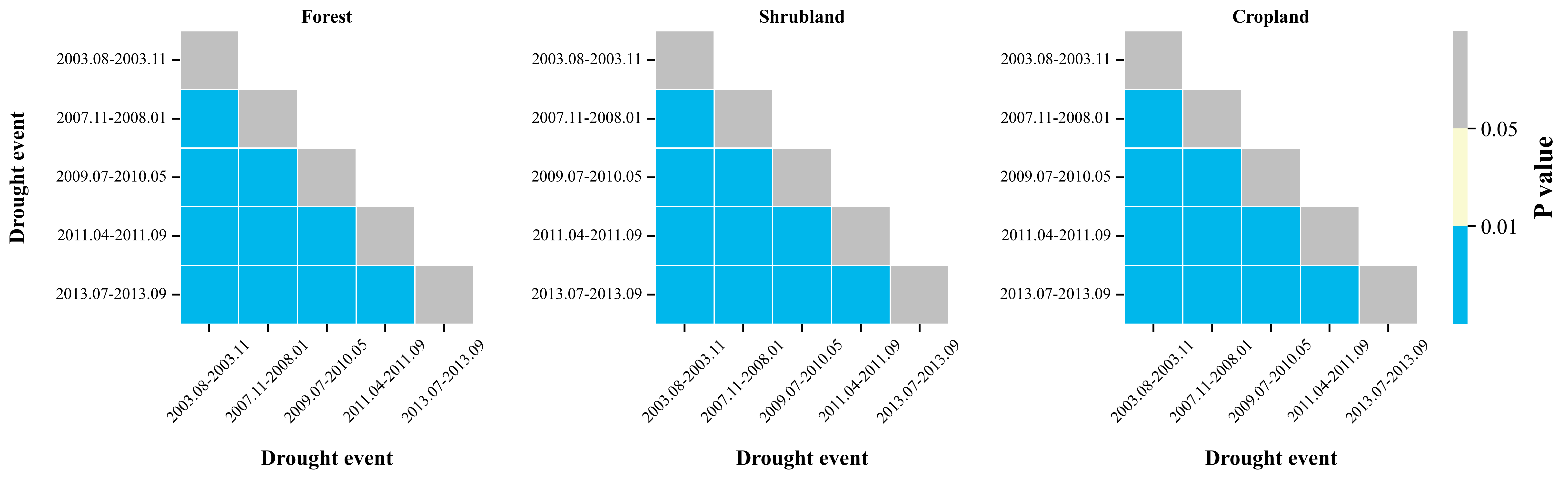


**Figure S4** Significance results of recovery period differences among drought events for the same land cover type (Kruskal-Wallis and Dunn tests). Blue indicates a highly significant difference (p < 0.01), light yellow indicates a significant difference (0.01 ≤ p < 0.05), and gray indicates a non-significant difference (p ≥ 0.05).


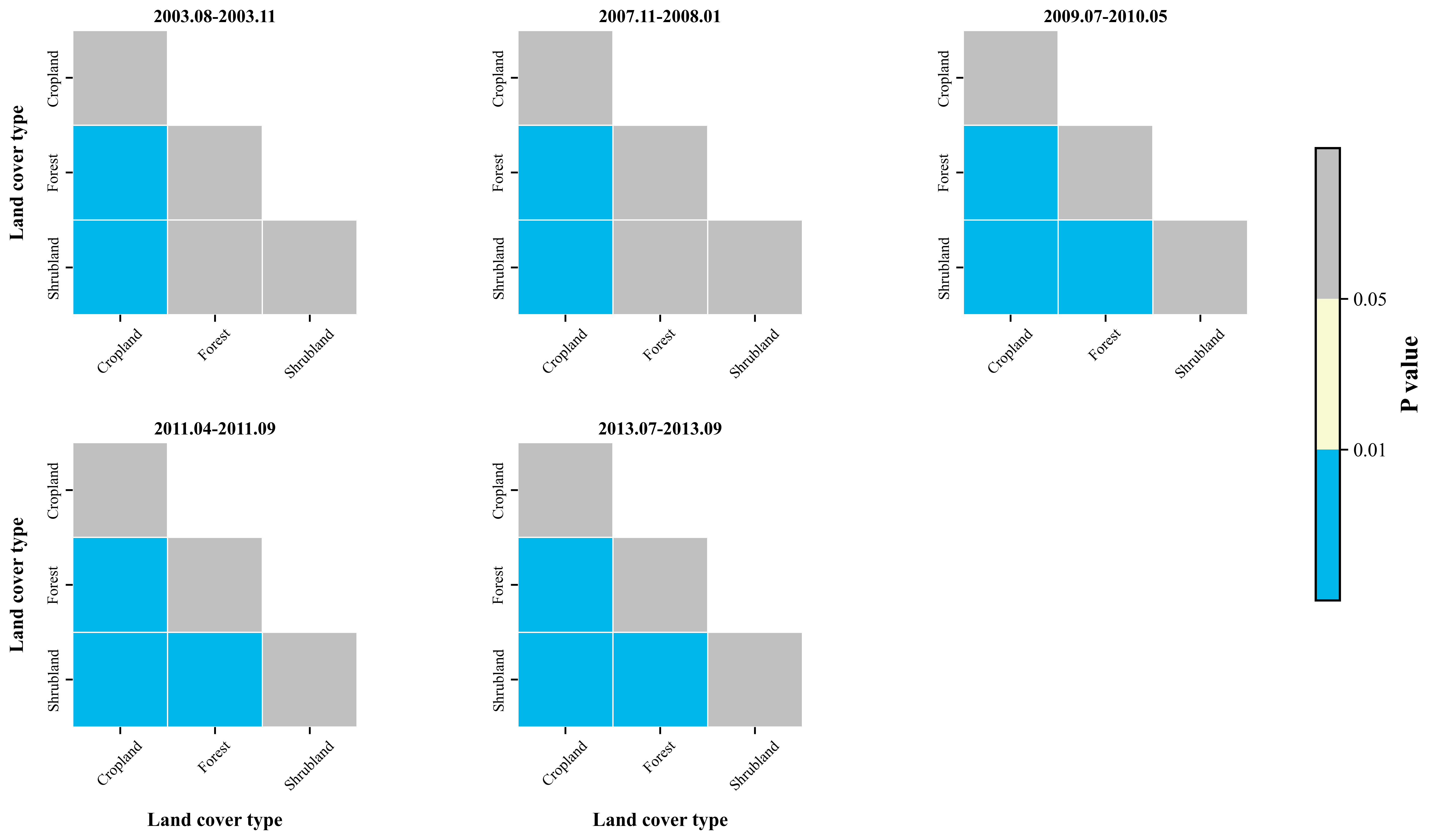


**Figure S5** Significance results of recovery period differences among land cover types within the same drought event (Kruskal-Wallis and Dunn tests). Blue indicates a highly significant difference (p < 0.01), light yellow indicates a significant difference (0.01 ≤ p < 0.05), and gray indicates a non-significant difference (p ≥ 0.05).


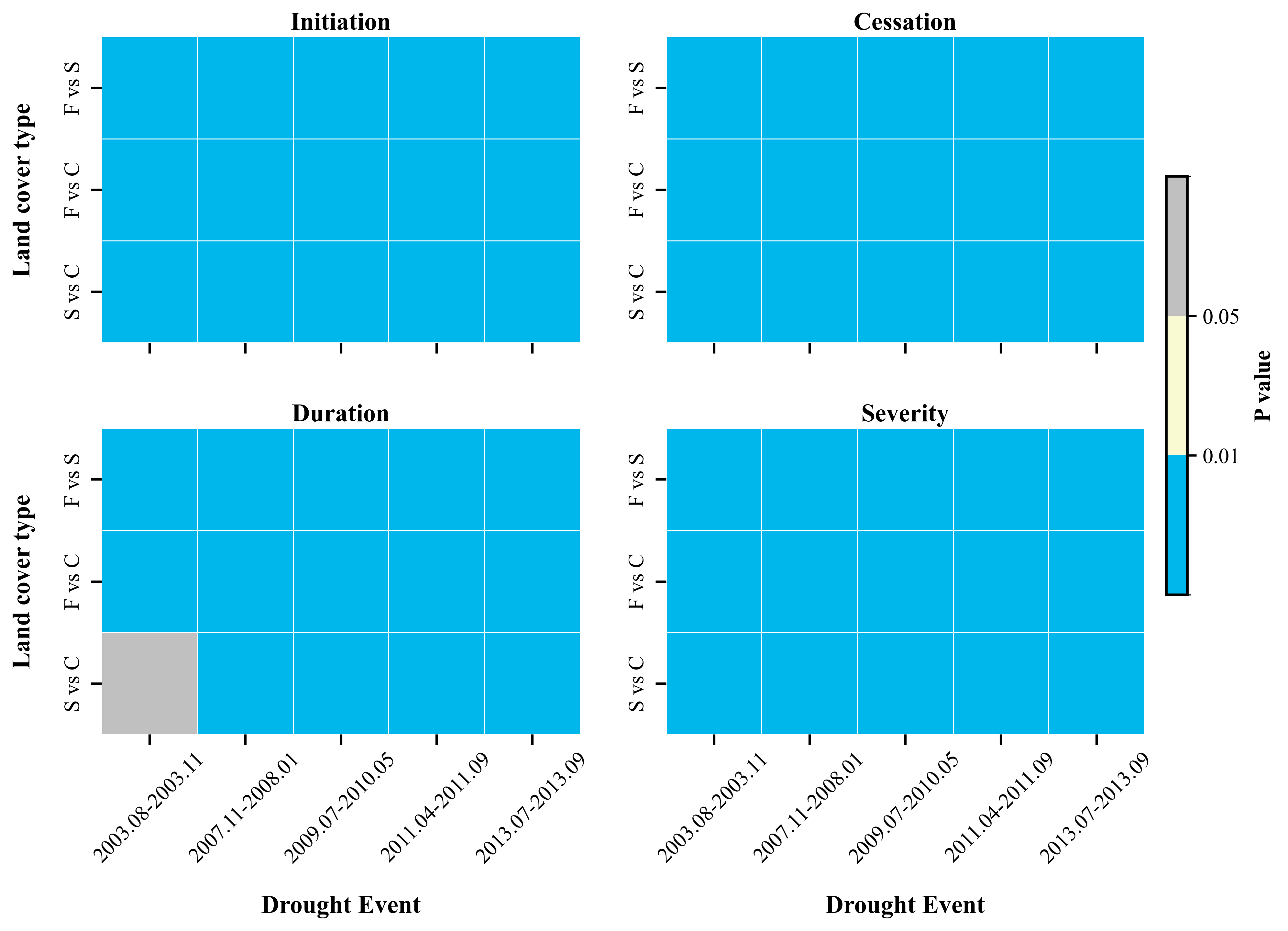


**Figure S6** Significance results of differences in drought characteristics (initiation, cessation, duration, and severity) among land cover types within the same drought event (Kruskal-Wallis and Dunn tests). Blue indicates a highly significant difference (p < 0.01), light yellow indicates a significant difference (0.01 ≤ p < 0.05), and gray indicates a non-significant difference (p ≥ 0.05).


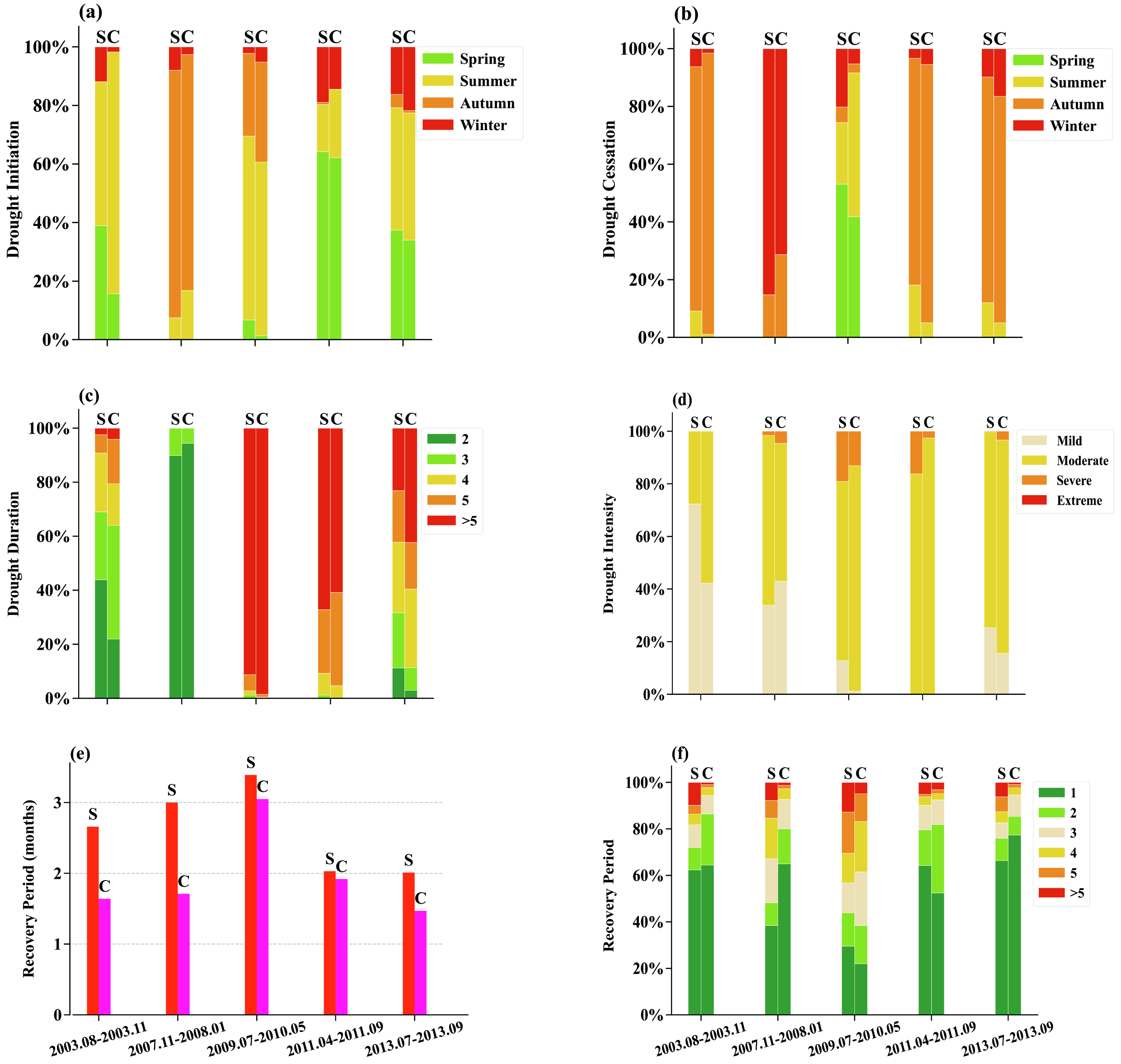


**Figure S7** Drought conditions across different land cover types, including (a) the percentage of drought initiation, (b) the percentage of drought cessation, (c) the percentage of drought duration, (d) the percentage of drought severity, (e) post-drought recovery time and (f) recovery duration percentage required by different land cover types. Here, S represents shrublands, and C represents croplands.
